# Efficacy of Mobile App–Based Dietary Interventions Among Cancer Survivors: Systematic Review and Meta-Analysis

**DOI:** 10.2196/65505

**Published:** 2025-07-31

**Authors:** Krista Ching Wai Chung, Naomi Takemura, Wendy Wing Tak Lam, Mandy Man Ho, Antoinette Marie Lee, Wynnie Yuen Yee Chan, Daniel Yee Tak Fong

**Affiliations:** 1 School of Nursing, University of Hong Kong, 5/F, HKUMed Academic Building, 3 Sassoon Road, Pokfulam, Hong Kong, China (Hong Kong), 852 39176645; 2 School of Nursing, Hong Kong Polytechnic University, Hong Kong, China (Hong Kong); 3 School of Public Health, University of Hong Kong, Hong Kong, China (Hong Kong); 4 Department of Psychology, University of Hong Kong, Hong Kong, China (Hong Kong); 5 School of Professional and Continuing Education, University of Hong Kong, Hong Kong, China (Hong Kong)

**Keywords:** mobile app, mobile phone, mHealth, cancer survivorship, dietary intervention, digital health intervention, behavior change, mobile health

## Abstract

**Background:**

The World Health Organization recommends that cancer survivors maintain a healthy diet and weight control to prevent cancer recurrence. Albeit a growing interest in using mobile apps for health promotion, there is a need for comprehensive evidence on the effects of mobile apps, particularly on dietary behaviors.

**Objective:**

This study aims to evaluate the efficacy, feasibility, and acceptability of mobile app–based dietary interventions among cancer survivors and explore the potential mobile app features worth incorporating.

**Methods:**

In this systematic review and meta-analysis, we searched Embase, Cochrane Library, PubMed, and Web of Science from inception to September 2023 without language restriction. We identified studies that used mobile apps for dietary interventions as a major module for cancer survivors. In addition, 2 independent reviewers screened the studies, extracted data, and assessed methodological quality using Cochrane’s risk of bias tools for randomized trials (RoB 2) and nonrandomized studies (ROBINS-I). A meta-analysis was conducted on body weight, BMI, nutritional outcomes, and quality of life using random-effects models.

**Results:**

Of the 2621 records identified, 22 studies involving 1204 cancer survivors were included. Notably, existing trials involved only breast and gastrointestinal cancer survivors. Preliminary evidence suggested that mobile app–based dietary interventions demonstrated a beneficial effect on energy intake (Hedges *g*=1.00, 95% CI 0.96-1.03) and weight changes (Hedges *g*=−0.43, 95% CI −0.45 to −0.41); as well as a potential to improve protein intake and quality of life among gastrointestinal cancer survivors. The usability, quality, and satisfaction of app use as measured by standardized questionnaires, including the System Usability Scale, the Mobile Application Rating Scale, and the Questionnaire for User Interface Satisfaction, were positive. While feedback messages and dietary goal setting were considered facilitators of mobile app use, concerns regarding the time required for app use and limited food logging options were raised.

**Conclusions:**

Our review found the preliminary efficacy, feasibility, and acceptability of mobile app–based dietary interventions for cancer survivors. However, study heterogeneity should be recognized. More trials are warranted to confirm the effectiveness of these interventions and explore any differences based on cancer types, staging, treatment statuses, the mode of communication with dietitians, and the engagement of family or caregivers. Existing mobile apps could maintain important features such as feedback messages and dietary goal setting while considering the incorporation of artificial intelligence-powered food recognition in food logging and cancer-specific dietary recommendations.

## Introduction

### Background

Cancer poses a significant global health concern, with a projected 77% increase in new cases by 2050, surpassing 35 million [1]. Early detection and treatment have resulted in a growing population of cancer survivors [2]. However, the journey of cancer survivorship presents various challenges, including side effects related to cancer treatments, pain, fear of cancer recurrence, financial concerns, impaired sleep, and cognitive functioning [2,3]. Furthermore, lifestyle behaviors such as unhealthy diet, sedentary behaviors, smoking, and alcohol consumption were associated with increased risks of cancer recurrence and overall mortality [4-7].

To address these challenges and reduce the risk of cancer recurrence, the World Health Organization and the World Cancer Research Fund International recommend cancer survivors adopt lifestyle behavioral modifications. These include maintaining a healthy weight, exercising regularly, having a healthy diet, and restricting the consumption of fast foods, red and processed meat, and alcohol [8,9]. Among these recommendations, dietary control for weight reduction and diet quality to ensure adequate nutritional intake are particularly important.

There has been mounting evidence suggesting the relationships between obesity and carcinogenesis. A meta-analysis of 203 observational studies, involving more than 6.3 million participants, concluded that obese patients with breast, colorectal, or prostate cancer experienced higher cancer mortality and relapse rates [10]. While the underlying mechanisms of carcinogenesis in obese individuals remain uncertain, it has been proposed that hyperinsulinemia, elevated BMI, and the overproduction and over-secretion of estrogen and adipokines by adipose tissues may trigger carcinogenesis [11]. Thus, implementing effective dietary control programs for reducing weight in obese cancer patients may help to mitigate mortality and recurrence risks.

In addition, diet quality plays a vital role, especially considering that the etiology of malnutrition among cancer survivors could be different from that in the general population. Specifically, while insufficient nutritional intake may generally be associated with anorexia or problems with oral intake, malnutrition among cancer survivors could also stem from catabolic metabolic derangements [12]. Compliance with dietary guidelines or evidence-based recommendations for better diet quality has been associated with improved survival outcomes among breast and colorectal cancer patients [13]. Therefore, all cancer survivors should undergo regular screening for malnutrition in accordance with the European Society for Clinical Nutrition and Metabolism guideline [12], and nutritional counseling should be provided throughout cancer survivorship [14]. It is important to prioritize adequate energy, protein, and fluid intake over weight management and dietary fiber intake for cancer survivors experiencing treatment-related symptoms and difficulties complying with general recommendations [14].

Traditionally, dietary interventions for cancer survivors have been delivered through face-to-face and telephone formats. A meta-analysis of 25 randomized controlled trials (RCTs) published in 2019 demonstrated the positive impact of traditional dietary interventions on fruit and vegetable intake as well as diet quality among cancer survivors who had completed active treatments [15]; and a network meta-analysis of 98 RCTs published in 2021 concluded that traditional dietary interventions, delivered alone or combined with exercise, were associated with greater reductions in BMI, waist circumferences, and weight compared to standard care among early-stage overweight and obese cancer survivors [16]. While traditional delivery of dietary interventions appears to be effective, it may limit the accessibility, adherence, and engagement of the intervention. The emergence of mobile health (mHealth) technology has revolutionized the provision of remote care for cancer survivors, enabling dietary intervention by alternative methods such as mobile apps, websites, and emails. In addition to synchronous care, which care is provided and received simultaneously, these platforms can facilitate the asynchronous delivery of dietary interventions that allow users to receive care at their convenience. With the growing interest in dietary interventions delivered by mobile apps in the recent decade, several studies had applied mobile apps with diverse features and examined their impact on a variety of outcomes, including anthropometric changes, dietary patterns, nutritional status, and quality of life (QoL) among cancer survivors. Although the existing systematic reviews by Gong et al [17] and Wang et al [18] evaluated the effects of mHealth apps on anthropometric changes, fruit and vegetable consumption, QoL, and fat intake among cancer survivors, they only included 2 studies with dietary interventions being delivered by mobile apps and were limited to English studies [17,18]. In addition, the systematic review by Wang et al [18] searched only 1 electronic database, potentially limiting the comprehensiveness of the findings. To our knowledge, there is no systematic review or meta-analysis specifically evaluating the impact of dietary interventions delivered by mobile apps, highlighting the need to synthesize the existing evidence on this body of research.

### Objective

Therefore, this study aims to perform a systematic review and meta-analysis to evaluate the effects of mobile app–based dietary interventions on anthropometric changes, nutritional outcomes, and QoL among cancer survivors. This review also aimed to explore the feasibility, acceptability, and potential features of mobile apps worth incorporating.

## Methods

This systematic review and meta-analysis was conducted following the Preferred Reporting Items for Systematic Reviews and Meta-Analyses (PRISMA) 2020 statement [19] (see Checklist 1). The study protocol was registered in the PROSPERO registry (CRD42023465641).

### Search Strategy

A comprehensive literature search was conducted from inception to September 19, 2023, in EMBASE, Cochrane Library, PUBMED, and Web of Science with no language restrictions. The search terms included (cancer OR oncology OR tumour OR tumor OR malignan*) AND (diet* OR nutrition* OR behavior* OR behavior*) AND (mobile app* OR mhealth OR smartphone app* OR mobile-assisted OR technology-supported OR app OR e-health). The search strategy for each database is listed in Multimedia Appendix 1.

### Eligibility Criteria

A study was eligible if it (1) evaluated a mobile health app with dietary intervention as the major component in either an intervention or a control group and (2) included patients diagnosed with cancer, regardless of cancer type, stage, and treatment status.

### Study Selection and Data Extraction

A total of 2 reviewers (KCWC and NT) independently screened the titles and abstracts of all retrieved records. After agreeing on a list of potentially eligible studies, they independently read the full texts to confirm the eligibility. Full texts of studies not written in English were translated into English using Google Translate. All inconsistencies were resolved by discussion with a third reviewer (DYTF). Reference lists of the identified studies were also read to determine if there were more relevant studies. Corresponding authors of the included studies were contacted when information on the study methods or results was unclear.

The outcomes of interest were body weight, BMI, QoL, dietary patterns, and nutritional status. Among the eligible studies, information regarding study design, settings, sample characteristics, descriptions of intervention and control, follow-up duration, attrition rates, outcomes of interest at baseline and follow-up time points, and acceptability in terms of quality, satisfaction, and usability of app use were extracted independently by 2 reviewers using a standardized form (KCWC and NT).

### Quality Assessment

The quality of RCTs was assessed using the Cochrane Collaboration’s Tool for Assessing Risk of Bias (RoB) 2.0, whereas that of single-arm trials and quasi-experimental studies was evaluated using Risk of Bias in Nonrandomized Studies (ROBINS-I) [20]. The evaluation was conducted independently by 2 reviewers (KCWC and NT). They also independently assessed the certainty of evidence of each meta-analysis result by the Grading of Recommendations Assessment, Development and Evaluation (GRADE) approach [21].

### Data Synthesis and Analysis

Outcomes of interest, common features, feasibility, and acceptability outcomes on which the included studies evaluated the mobile apps were first identified. To obtain the overall effect size of the outcomes of interest, their postintervention means and SDs were extracted to estimate the standardized mean difference (SMD), specifically Hedges *g*, with 95% CIs. Hedges *g* was calculated to standardize the study intervention effects of different studies based on a uniform scale [20]. According to the Cochrane Handbook, SMDs of 0.2, 0.5, and 0.8 represent small, moderate, and large effects, respectively [20]. For each outcome of interest, meta-analysis was performed when at least 2 RCTs measured the same outcome of interest. Thus, only RCTs with at least two of them reporting the same outcome of interest were included in the quantitative synthesis. Random-effects models were adopted, given the heterogeneous mobile app features and intervention durations. Heterogeneity across studies was then assessed based on *I*^2^ statistics [20]. Depending on the magnitude and direction of effects and the strength of evidence for heterogeneity, approximately an *I*^2^ statistic between 0% and 40% indicates low heterogeneity; between 30% and 60% conveys moderate heterogeneity; between 50% and 90% represents substantial heterogeneity; and 75% and 100% implies considerable heterogeneity [20]. Due to the limited number of available trials, funnel plots to assess publication bias and sensitivity analyses could not be performed. All meta-analyses were conducted using RStudio (version 4.3.1; Posit). All significance tests were 2-tailed, and a *P* value of <.05 was considered statistically significant.

## Results

### Study Selection

**Figure 1.**
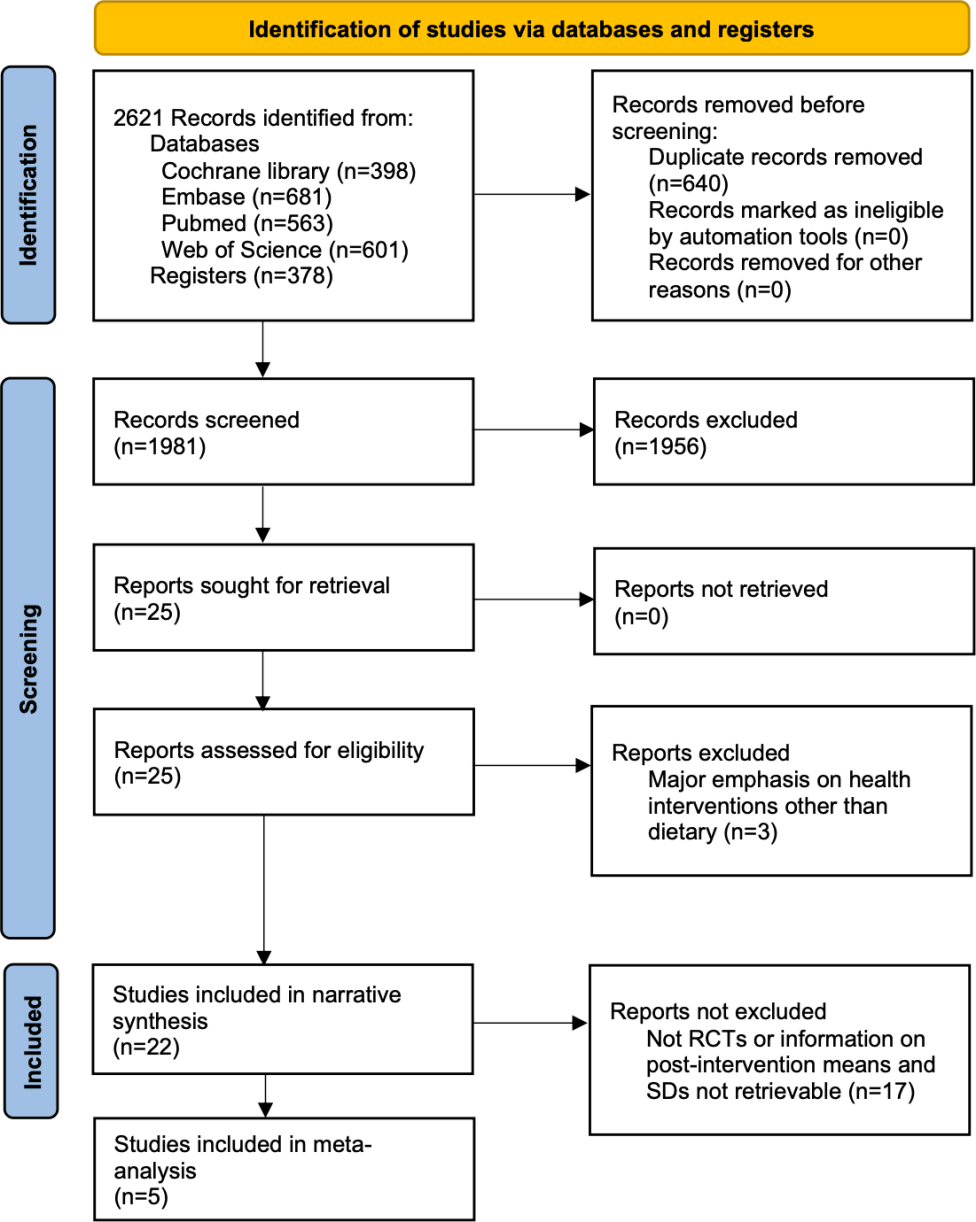
PRISMA (Preferred Reporting Items for Systematic Reviews and Meta-Analyses) flow diagram for included studies. RCT: randomized controlled trial.

Figure 1 shows the PRISMA flow diagram. The search identified 2621 records from 4 databases. No additional record was found from citation searching. After removing 640 duplicate records and screening the titles and abstracts, 25 studies remained. Their full texts were retrieved and reviewed. Ultimately, 22 studies, comprising 2 in Korean and the remaining in English, met the predetermined eligibility criteria and were included in the review.

### Study and Participants’ Characteristics

Of the 22 included studies, 8 were RCTs, 3 were quasi-experimental studies, and 11 were single-arm trials. Their study characteristics are summarized in Table 1 and in Multimedia Appendix 2. The studies were published between 2015 and 2023 and conducted in South Korea (n=8), the United States (n=7), China (n=3), Australia (n=1), Germany (n=1), Iran (n=1), and Spain (n=1). The sample size ranged from 16 to 127, amounting to a total of 1204 participants. The participants’ mean age ranged between 15 and 68 years. A total of 9 (45%) studies considered only breast cancer [22-30], whereas 1 study (5%) involved breast and/or endometrial cancer [31], followed by 8 (36%) on gastrointestinal (GI) cancer [32-39], 2 (9%) on leukemia or lymphoma [40,41], and 1 (5%) on lung cancer [42]. There was 1 study that considered a mix of different cancer types [43]. A total of 10 (45%) studies involved cancer survivors at nonmetastatic stages (0-III) [23,24,26-32,38], and 11 (50%) studies included cancer survivors who had either completed [22,23,25,27-30,38,41] or no longer required active cancer treatments [31,40].

**Table 1.** Study characteristics.

Author (year)	Country	Study design	Sample size, n	IG^a^, n	CG^b^, n	Females, n (%)	Age (years), mean (SD)	Cancer type, staging, and treatment status	Intervention	Control	Intervention + follow-up duration (weeks)
Allicock et al (2021) [22]	United States	RCT^c^	22	13	9	22 (100)	52 (9)	Breast, NR^d^, completed (excluding Herceptin and endocrine therapies).	Ecological momentary assessments by Project CHAT^e^ app + waist-worn accelerometers + newsletter about healthy diet and physical activity.	Waist-worn accelerometers + newsletter.	4 + 8
Baik et al (2020) [23]	United States	RCT	80	40	40	80 (100)	53 (11)	Breast, 0-III, completed (excluding endocrine therapy).	Psychoeducation and self-management program by My Guide app + telecoaching.	Healthy lifestyle promotion by My Health app + telecoaching.	6 + 8
Cairo et al (2020) [24]	United States	Quasi-experimental study	127	66	61	127 (100)	51^a^ (8) and 57^b^ (10)	Breast, 0-III, NR.	Coach-guided dietary interventions by Vida app + printed survivorship care plan.	Printed survivorship care plan + self-guided “toolkit”.	24 + 48
Cheng et al (2020) [32]	China	Single-arm trial	20	20	NA^f^	2 (10)	62 (7)	Esophageal, I-III, Post-esophagectomy.	Dietary, exercise, and mental support by WeChat app in addition to standard postoperative care.	NA	12 + 12
Choi et al (2020) [25]	South Korea	RCT	50	25	25	50 (100)	49 (8)^a^ and 50 (10)^b^	Breast, NR, completed.	Nutritional management program delivered by efilcare R app.	Continued daily life as usual.	4 + 4
Chow et al (2021) [40]	United States	RCT	41	24	17	20 (49)	45 (20-55)^g^	Hematologic, NR, in remission but not receiving.	Diet and physical activity promotion by Healthwatch360 app and Fitbit app + Fitbit Flex wristband + telephone-based consultation.	Given access to Fitbit tracker and HealthWatch360 app, but did not receive usage reminders or guidance.	16 + 24
Fuemmeler et al (2020) [41]	United States	Single-arm trial	16^h^	16	NA	7 (40)	15 (2)	Hematologic, NR, completed.	Healthy diet and physical activity promotion by Mila Blooms app + pedometer + graphic novel + one brief motivational phone interview + newsletters and educational materials.	NA	8 + 8
Huggins et al (2022) [33]	Australia	3-arm RCT	111	38^i^ and 36^j^	37	37 (33)	67 (10)^j^ and 68 (10)^i^	Upper gastrointestinal (esophageal, gastric, and pancreatic), all clinical staging, just received emergent surgery and pending curative cancer treatments.	Telephone group: nutritional assessment and education by phone + telephone reviews; myPace app group: nutritional assessment and education + electronic reviews.	Usual care	18 + 48
Jiang et al (2023) [34]	China	RCT	24	12	12	8 (33)	55 (10)	Gastric, I-IV, post-discharged after gastrectomy.	Dietary intervention by iNutrition applet + telephone dietary consultations.	Usual care	12 + 12
Keum et al (2021) [35]	South Korea	RCT	40	20	20	15 (38)	49 (8)^a^ and 50 (10)^b^	Pancreatic, all clinical staging, receiving chemotherapy.	Dietary intervention to promote caloric intake by Noom app	No access to Noom	12 + 12
Lim et al (2023) [26]	South Korea	Single-arm trial	29	29	NA	29 (100)	43 (8)	Breast, 0-IIB, just received surgical treatment and pending chemo-, radio-, or hormonal therapy.	Tailored post-surgery self-management by Breast Cancer by Second Doctor app + smart band.	NA	48 + 48
Lozano-Lozano et al (2019) [27]	Spain	Single-arm trial	80	80	NA	80 (100)	52 (9)	Breast, I-IIIA, completed	Energy balance-focused dietary interventions by BENECA^k^ app	NA	8 + 8
McCarroll et al (2015) [31]	United States	Single-arm trial	50	50	NA	50 (100)	58 (10)	Breast and/or endometrial, I-II, not requiring treatment with no sign of recurrence.	Weight loss and dietary modifications by “beta” health care provider version LoseIt! app + weight tracking by Bluetooth scale.	NA	4 + 4
Orlemann et al (2018) [43]	Germany	Quasi-experimental study	24	12	12	24 (62)	NR	Mixed (67% GI cancer), NR, and NR.	Food record by OncoFood app + paper nutritional documentation + nutrition counseling.	Usual care	4 + 4
Park et al (2019) [28]	South Korea	Quasi-experimental study	71	36	35	71 (100)	52 (4)^a^ and 53 (6)^b^	Breast, 0‐3A, completed active treatments and receiving outpatient follow-up.	Promotion of healthy behaviors (eg, diet and exercising) and peer support by MyFitnessPal and RunKeeper apps + face-to-face health education and peer support group meetings.	Usual care	12 + 24
Salmani et al (2022) [36]	Iran	Single-arm trial	17	17	NA	7 (41)	57 (17)	Colorectal, NR, receiving.	Cancer self-management by the “Colorectal Cancer Along” app.	NA	2 + 2
Seo et al (2021) [29]	South Korea	Single-arm trial	20	20	NA	20 (100)	51 (7)	Breast, I-III, completed.	Promotion of healthy lifestyle (eg, diet and exercising) by “Health for You” app.	NA	2+ 2
Soh et al (2018) [37]	South Korea	Single-arm trial	203	203	NA	75 (37)	NR^l^	Gastric or colon, NR, received surgery or receiving chemotherapy (50.2% receiving palliative care).	Support for nutrition, QoL^m^, and rehabilitation by multidisciplinary mobile care system, Life Manager app + anthropometric measurements.	NA	12 + 12
Stubbins et al (2018) [30]	The United States	Single-arm trial	33	33	NA	33 (100)	57 (9)	Breast, I-III, completed.	Daily record of diet and physical activity + real-time communication with dietitians by MOCHA^n^ app.	NA	4 + 4
Wang et al (2022) [38]	China	RCT	60	30	30	25 (45)	69 (7)^a^ and 68 (9)^b^	Colorectal, I-III, completed.	Dietary consultation and adjustment of dietary plans by dietitians by WeChat app-based video calls + nutritional status assessments during two home visits.	Routine care (telephone follow-up assessments on disease-related symptoms + lifestyle advice + mental rehabilitation).	24 + 24
Yang et al (2021) [39]	South Korea	Single-arm trial	36	36	NA	0 (0)^o^	59^g^^,^^p^	Esophageal, all clinical, receiving neoadjuvant concurrent chemoradiotherapy.	Health coaching interventions to prevent malnutrition and excessive muscle loss by Noom app.	Retrospective review by Yoon et al (2020) among subjects who underwent operation and received usual care without Noom use.	8 + 8
Yang et al (2022) [42]	South Korea	Single-arm trial	50	50	NA	22 (44)	58 (12)	Lung, I-IV, receiving outpatient chemotherapy treatments or paying routine outpatient visits after lung resection surgery.	Tailored diet and exercise program by Smart After-Care app + self-monitoring devices (eg, sphygmomanometer, finger pulse oximeter, and digital spirometer) + telephone health counseling.	NA	12 + 12

a IG: intervention group.

b CG: control group.

c RCT: randomized controlled trial.

d NR: not reported.

e CHAT: Creating Healthy Actions through Technology.

f NA: not available.

g Age reported as median (IQR).

h Parent-adolescent dyads.

i Telephone intervention group.

j myPace app intervention group.

k BENECA: The Energy Balance on Cancer.

l 35.5% in their 50s.

m QoL: quality of life.

n MOCHA: Methodist Hospital Cancer Health Application.

o All participants were male.

p IQR not reported.

### Characteristics of Dietary Interventions and Features of Mobile Apps

A total of 8 (36%) studies used mobile apps as the sole delivery means of dietary interventions [25,27,29,30,33,35,36,39]. Other studies used additional delivery means such as telephone-based consultations [23,24,34,40,42] and face-to-face health educational classes plus support group meetings [28]. Among all the included studies, 10 studies (45%) had app users maintain regular interaction with dietitians [25,30,33-35,38,43] or coaches [24,39,41]. Both the intervention period and follow-up duration of the 22 studies ranged between 1 to 12 months, with a median of 2.5 months.

Among the 22 included studies, 2 applied the WeChat app (a messaging and social media app; Tencent), 2 used the Noom app (a weight management and behavior change app), and the remaining 18 studies used different mobile apps, summing up a total of 20 different mobile apps. These mobile apps incorporated different intervention components. A total of 5 (23%) studies used mobile apps that exclusively focused on diet [25,33,34,38,43]. However, 10 (45%) studies adopted apps with additional components on physical activity [22,27,28,30,31,35,39-42], while the remaining 7 (32%) studies additionally incorporated other health-related components, including cancer symptom management, mental health support, medication, and pain management [23,24,26,29,32,36,37]. A total of 8 (36%) studies incorporated a theoretical framework to develop or deliver interventions, which included the Social Cognitive Theory (n=4) [22,27,28,31], the Self-Determination Theory (n=2) [40,41], the Health Action Process Approach theory (n=1) [34], and the Supportive Accountability Framework (n=1) [33].

A total of 6 common features of the 20 mobile apps are identified and summarized (see Table 2). The most common feature was self-diet monitoring [22,24-37,39-41,43], followed by dietary goals setting by health care professionals, research staff, or mobile app users [24,27-31,33,34,36,37,40,41,43], feedback messages (messages based on self-diet monitoring data [22,24,25,27,31,32,35,41], motivational messages [22,25,31], and replies to mobile app users’ questions [30,32,34,35,38,39]), self-body weight monitoring [24,29,31,33-35,37,39,43], personalized dietary management planning either developed by dietitians, research staff, or co-developed by dietitians and mobile app users [25,33,34,38,42], and social platforms to facilitate peer support [28,32,34,41].

**Table 2.** Summary of adopted mobile app features.

Mobile app features	Studies, n (%)
Self diet monitoring	19 (86)
Dietary goals setting	13 (59)
Feedback messages	12 (55)
Self body weight monitoring	9 (41)
Personalized dietary management planning	5 (23)
Social platform	4 (18)

### Methodological Quality

The risk of bias in all RCTs and nonrandomized trials is summarized in Multimedia Appendix 3. All RCTs were rated as having “some concerns” [22,23,25,33,34,38,40] due to the approach of measuring self-reported outcomes. Among the nonrandomized trials, 3 studies were identified as having serious bias [24,26,28], while the risk of bias in 1 study could not be determined due to insufficient information [43]. One study had a serious bias due to confounding and selection bias, as it did not control for the prognostic factor age that showed significant baseline difference, and its recruitment of curative-intent cancer survivors may have included participants with a higher level of health consciousness [24]. Another study had a serious bias due to substantial missing data, as it had 42% of participants dropping out before the start of the intervention and a high attrition rate of 61% [28]. The other study had serious measurement bias due to the use of non-validated instruments [26].

### Usability, Quality, and Satisfaction of Mobile App Use

A total of 9 of the 20 mobile apps were evaluated for their usability, quality, or satisfaction, which involved breast, GI, and lung cancer survivors. Their evaluation results are summarized in Multimedia Appendix 4. In addition, 5 of the 9 apps were evaluated using standardized questionnaires, including the 10-item System Usability Scale (SUS; n=2) [30,34], the 23-item Mobile Application Rating Scale (MARS) and its 26-item user version (n=2) [27,29], and the 27-item Questionnaire for User Interface Satisfaction (QUIS; n=1) [36]. The other 4 apps were rated using nonstandardized questionnaires assessing satisfaction and usability [25,26,37,42].

Only 2 studies evaluated the usability of their apps using the SUS [30,34], whereas another study rated its app using a self-developed scale [26]. With a mean total SUS score above 70 considered acceptable and with good adjective ratings [44], the MOCHA (Methodist Hospital Cancer Health Application) app was rated with a mean total SUS score of 77.4 (SD not reported) among stage I to III breast cancer survivors [30], and the iNutrition applet was rated with a mean total SUS score of 77.27 (SD 10.69) among postgastrectomy cancer survivors [34]. The Second Doctor app was evaluated based on a self-developed 14-item usability scale and received a mean total usability score of 80.2 out of 100 among breast cancer survivors [26].

Only 2 studies examined the quality of their apps using the MARS and the user version of the MARS (uMARS) [27,29]. Both scales rate app quality based on domains including engagement, functionality, aesthetics, information, app subjective quality, and perceived app use impact, with individual items rated on a 5-point scale, in which “1” indicates inadequate and “5” represents excellent [45,46]. The BENECA and “Health for You” apps received a mean total MARS and uMARS score of 3.71 (SD 0.47) and 3.60 (SD 0.69), respectively, among stage I to III breast cancer survivors [27,29].

Regarding satisfaction, only 1 app was rated using the QUIS [36], while 4 other apps were evaluated by nonstandardized self-developed questionnaires [25,26,37,42]. The “Colorectal Cancer Along” app was assessed on user satisfaction using QUIS on a 10-point Likert scale, which assesses specific domains, including overall reaction to the app, screen design and layout, terminology and application information, learnability, and app features [36]. The higher the score, the greater the level of satisfaction with the user interface [36]. Specifically, the QUIS score of this app for the “overall reaction to the app” domain was 7.94 (SD 1.38) among colorectal cancer survivors receiving active cancer treatments [36]. Among the other 4 apps, which involved breast, GI, and lung cancer survivors, a higher score on the non-standardized questionnaires indicates a higher level of app use satisfaction [25,26,37,42]. The mean total satisfaction scores of the efilcare R app (LifeSemantics) and the Life Manager app (LifeSemantics) were between 3.93 and 4.2 out of 5 [25,37], and that of the Second Doctor app was 22.4 out of 30 [26]. While the mean total score was not reported, 88% of the Smart After-Care app users rated app use satisfaction as “very good” or “good” [42].

### Facilitators and Barriers to App Use

A total of 5 mobile apps were evaluated qualitatively [24,26,27,40,43]. Common facilitators of app use included the provision of educational information [27,40], feedback [27,40], motivational messages [40], and setting and tracking of nutritional goals [43]. On the other hand, barriers to app use included concerns about the amount of time committed to app use [24,26,43], limited food diary entry options for assessing food intake [26,27], perceived ineffectiveness of action plans for inducing behavioral changes [40], challenges in adopting the app’s advice in real-life situations [40], and the lack of concrete meal plan to guide dietary choices [26].

### Effect on Anthropometric Measurements

**Figure 2.**
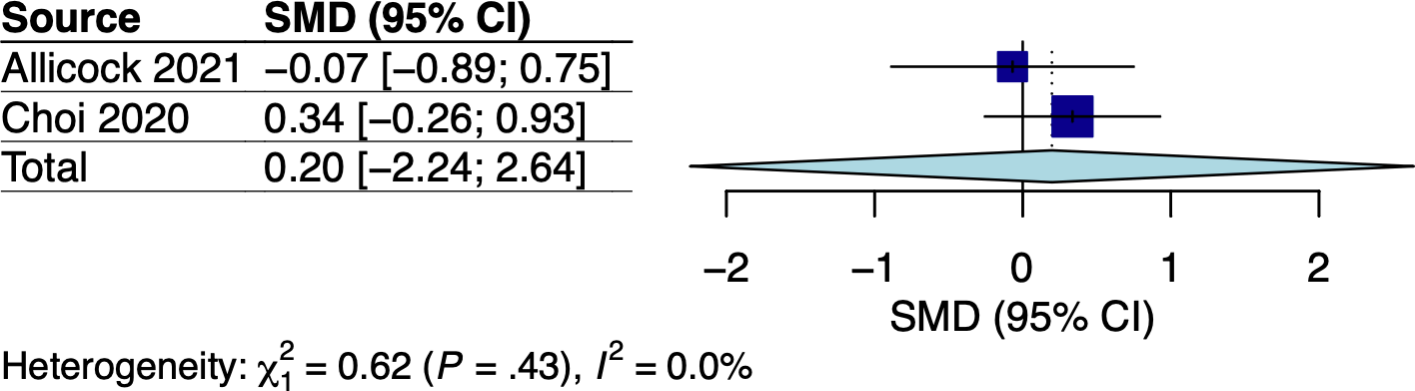
Meta-analysis results for body weight among breast cancer survivors [22,25]. SMD: standardized mean difference.

**Figure 3.**
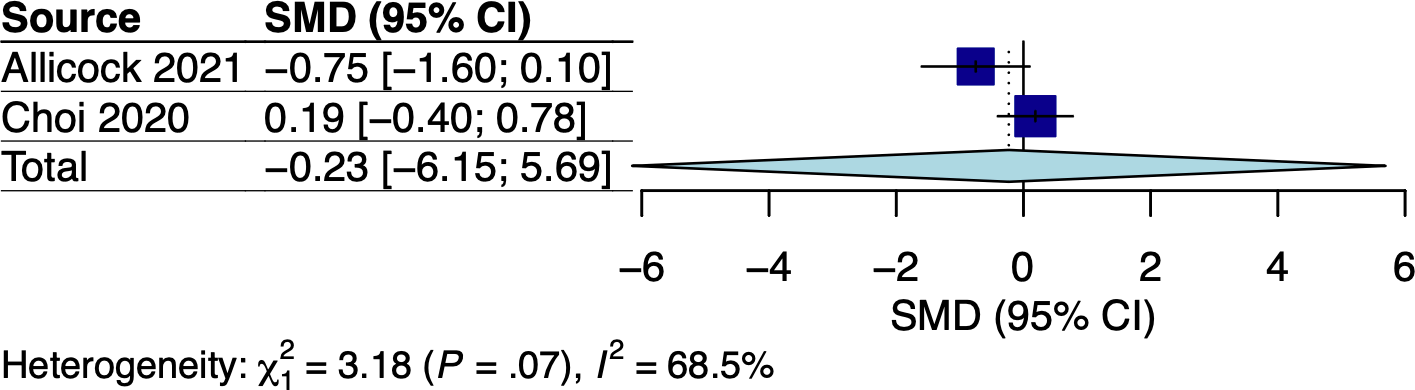
Meta-analysis results for BMI among breast cancer survivors [22,25]. SMD: standardized mean difference.

**Figure 4.**
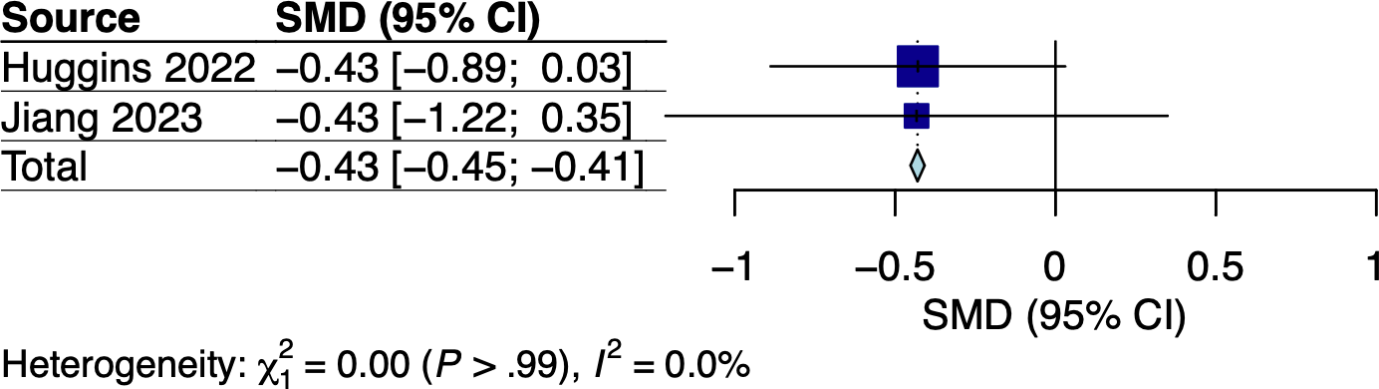
Meta-analysis results for body weight among gastrointestinal (GI) cancer survivors [33,34]. SMD: standardized mean difference.

**Figure 5.**
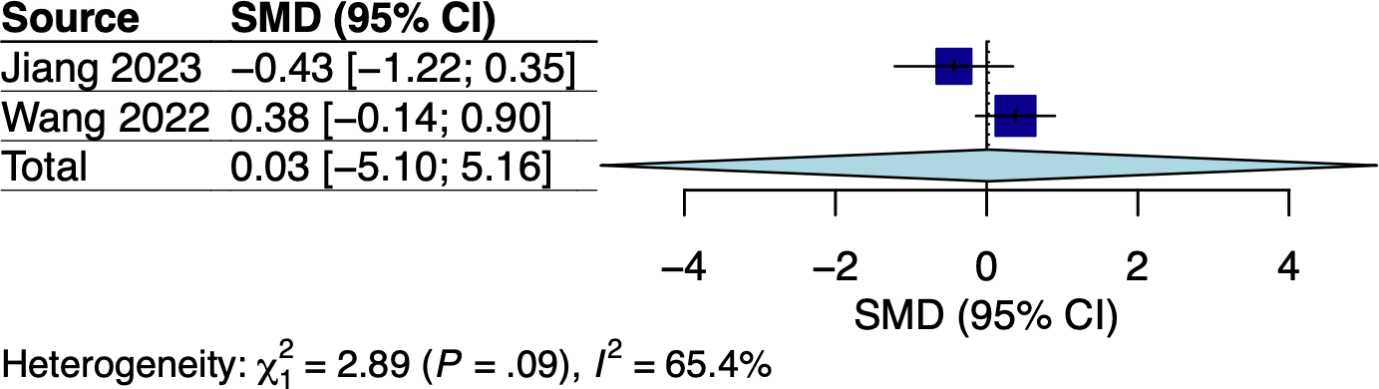
Meta-analysis results for BMI among gastrointestinal (GI) cancer survivors [34,38]. SMD: standardized mean difference.

A total of 5 RCTs assessed body weight and/or BMI, which involved breast and GI cancer survivors receiving dietary interventions that did not specify body weight goals [22,25,33,34,38]. The corresponding pooled effects are shown in Figures 2-5. For breast cancer survivors, their mobile app–based dietary interventions neither showed statistically significant effects on weight changes (Hedges *g*=0.20, 95% CI −2.24 to 2.64; *P*=.49; *I*^2^=0%; see Figure 2) nor on BMI (Hedges *g*=−0.23, 95% CI −6.15 to 5.69; *P*=.71; *I*^2^=69%; see Figure 3) compared to the control groups (see Table 1). For GI cancer survivors, meta-analysis showed a significant effect on weight changes (Hedges *g*=−0.43, 95% CI −0.45 to −0.41; *P*=.003; *I*^2^=0%; see Figure 4), but not on BMI (Hedges *g*=0.03, 95% CI −5.10 to 5.16; *P*=.96; *I*^2^=65%; see Figure 5) compared to control groups (see Table 1). The certainty of evidence for body weight using the GRADE approach was low for breast cancer and high for GI cancer survivors, respectively (see Multimedia Appendix 5). The certainty of evidence for BMI based on the GRADE approach was very low for both breast and GI cancer survivors (see Multimedia Appendix 5).

Among these 5 RCTs, only 1 study reported a baseline mean BMI that exceeded the overweight or obese threshold [22]. Another study did not report the baseline mean BMI [33]. The remaining 3 studies reported baseline mean BMIs within the normal range [25,34,38], although one of these studies only provided BMI at follow-up, without body weight information [38]. Subgroup analyses of patients with a normal baseline BMI showed insignificant effects compared to control groups for both body weight (Hedges *g*=0.002, 95% CI −4.84 to 4.85; *P*≥.99; I^2^=55%) and BMI (Hedges *g*=0.14, 95% CI −0.80 to 1.08; *P*=.59; *I*^2^=27%).

For nonrandomized trials, 3 studies conducted among overweight or obese breast cancer survivors [24,27,31] and another study involving esophageal cancer survivors [32] reported significantly reduced weight and BMI.

### Effect on Quality of Life

**Figure 6.**
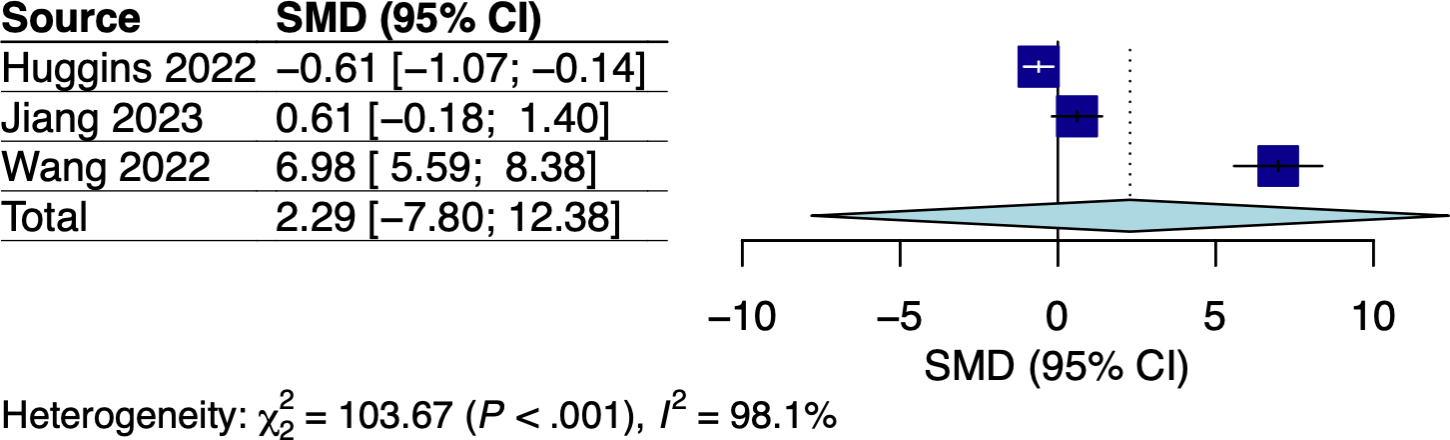
Meta-analysis results for quality of life among gastrointestinal (GI) cancer survivors [33,34,38]. SMD: standardized mean difference.

A total of 4 RCTs that involved GI cancer survivors and 3 single-arm trials which involved breast, esophageal, and lung cancer survivors reported QoL using the global health status domain of the European Organization for the Research and Treatment of Cancer Quality of Life Questionnaire (EORTC QLQ-C30) [27,32-35,38,42]. Since the mean global health status scores in the RCT by Keum and colleagues [35] were not reported, only 3 RCTs were included in our pooled analysis, with the corresponding pooled effect shown in Figure 6. Mobile app–based dietary interventions did not show an effect on QoL outcomes with substantial heterogeneity (Hedges *g*=2.29, 95% CI −7.80 to 12.38; *P*=.43; *I*^2^=98%; see Figure 6). However, meta-regression or subgroup analysis was not possible due to the limited available trials. The certainty of evidence using the GRADE approach was very low (see Multimedia Appendix 5). Among the single-arm trials, significantly improved within-group global health status scores were reported among breast and lung cancer survivors [27,42], whereas no improvement was reported among esophageal cancer survivors [32].

### Effect on Dietary Intake and Nutritional Status

#### Energy and Protein Intake

**Figure 7.**
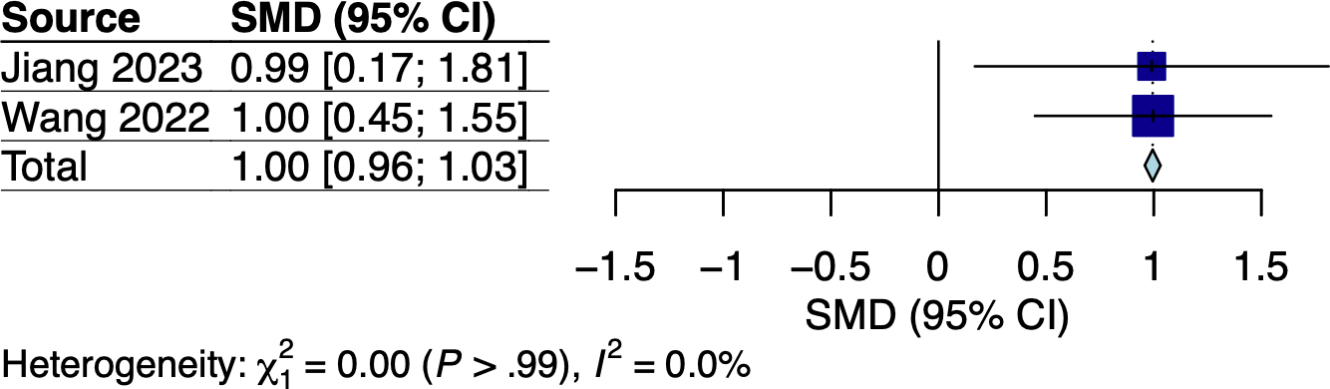
Meta-analysis results for energy intake among gastrointestinal (GI) cancer survivors [34,38]. SMD: standardized mean difference.

**Figure 8.**
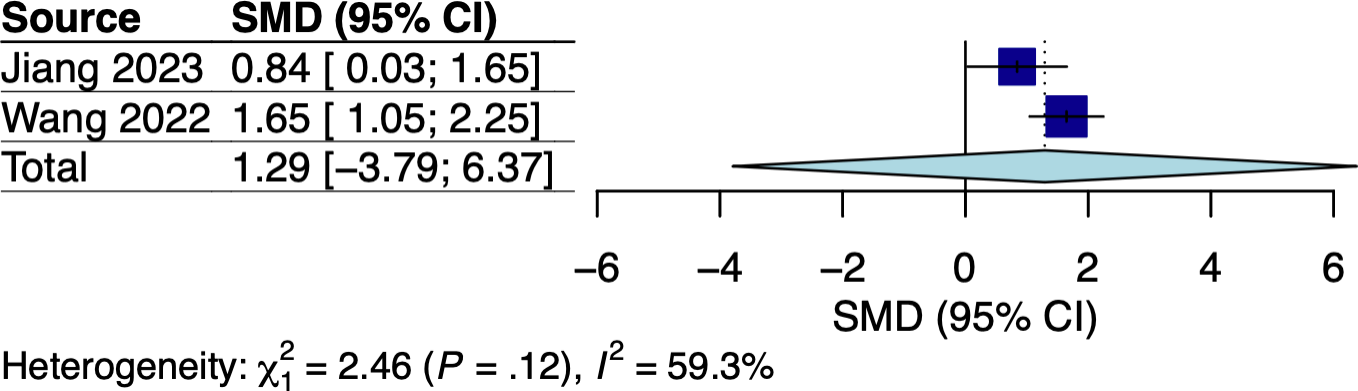
Meta-analysis results for protein intake among gastrointestinal (GI) cancer survivors [34,38]. SMD: standardized mean difference.

A total of 2 RCTs measured caloric and protein intake based on 24-hour dietary recall questionnaires among colorectal and gastric cancer survivors receiving dietary interventions tailored to individuals’ nutritional requirements [34,38], with the respective pooled effects shown in Figures7  8. Meta-analysis showed significant effects of mobile app–based interventions on energy intake (Hedges *g*=1.00, 95% CI 0.96-1.03; *P*=.002; *I*^2^=0%; see Figure 7). Nonetheless, mobile app–based interventions had no significant effect on protein intake with moderate heterogeneity (Hedges *g*=1.29, 95% CI −3.79 to 6.37; *P*=.19; *I*^2^=59%; see Figure 8). The certainties of evidence for energy and protein intake using the GRADE approach were high and moderate, respectively (see Multimedia Appendix 5).

#### Fast Food Intake

**Figure 9.**
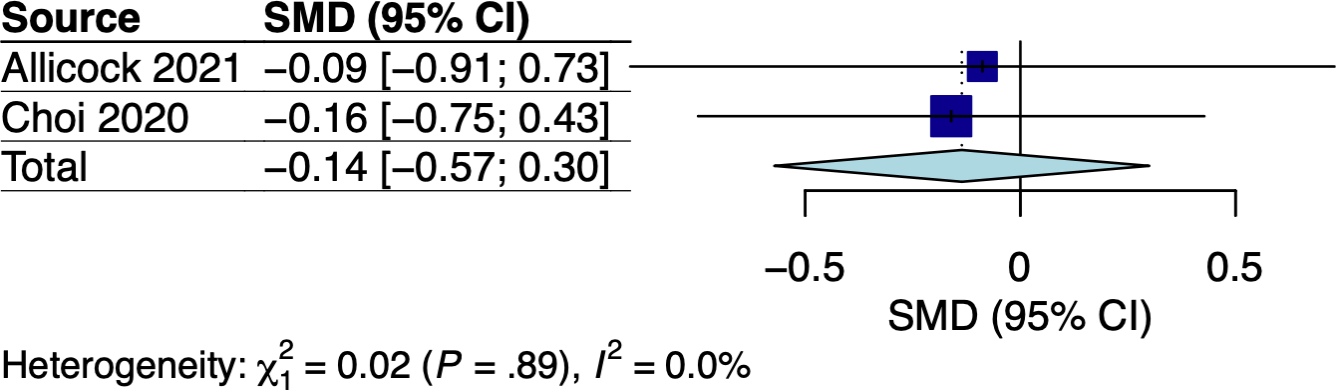
Meta-analysis results for fast food intake among breast cancer survivors [22,25]. SMD: standardized mean difference.

A total of 2 RCTs measured fast food intake using 24-hour recall questionnaires and food frequency questionnaires among breast cancer survivors [22,25], with the respective pooled effect shown in Figure 9. Mobile app–based dietary interventions had no significant effect on fast food intake (Hedges *g*=−0.14, 95% CI −0.57 to 0.30; *P*=.16; *I*^2^=0%; see Figure 9). The certainty of evidence using the GRADE approach was low (see Multimedia Appendix 5).

#### Vegetable and Fruit Intake

Only 2 of the 4 studies that assessed vegetable and/or fruit intake [22,25,28,41] based on 24-hour recall questionnaires and food frequency questionnaires reported significant between-group differences [25,28]. In the RCT by Choi et al [25], breast cancer mobile app users consumed significantly more weekly vegetables and fruit than the control group (see Table 1). Another quasi-experimental study by Park et al [28] also showed greater vegetable consumption among breast cancer survivors compared to the control group (see Table 1).

#### Sugar Intake

An RCT and a single-arm trial measured sugar intake using food frequency questionnaires among hematologic cancer survivors [40,41]. Neither significant between-group changes in sugar intake nor significant within-group changes in sugary drink consumption were reported [40,41].

#### Nutritional Status

**Figure 10.**
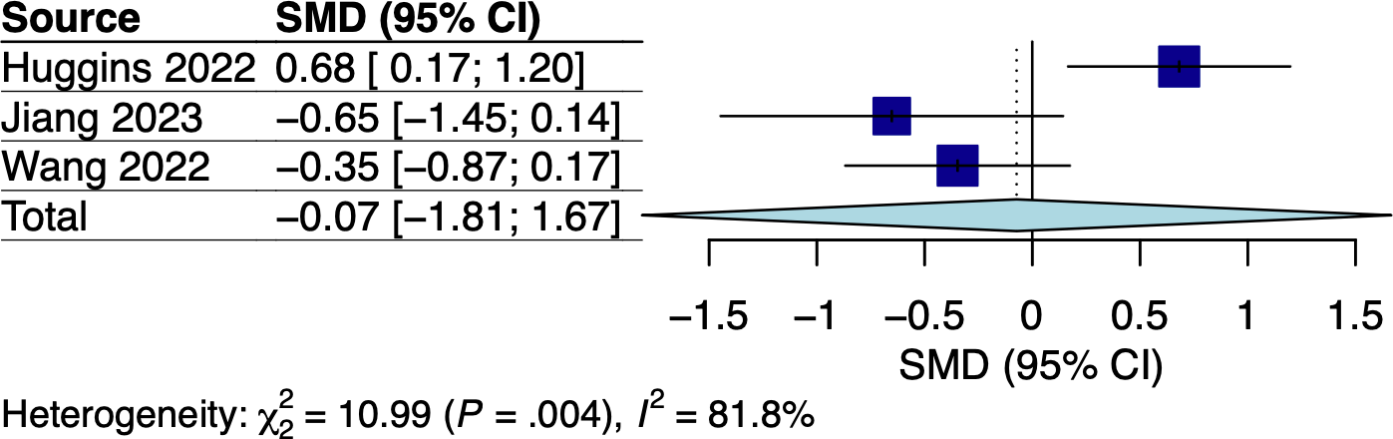
Meta-analysis results for nutritional status among gastrointestinal (GI) cancer survivors [33,34,38]. SMD: standardized mean difference.

A total of 4 RCTs [33-35,38] and 1 single-arm trial [47] used the Scored Patient-Generated Subjective Global Assessment (PG-SGA) and the prognostic nutritional index (PNI) to assess nutritional status. Given Keum et al [35] did not report the mean PG-SGA scores, we included only 3 RCTs in the pooled analysis, with the corresponding pooled effect shown in Figure 10. Meta-analysis did not show a significant effect on nutritional status among GI cancer survivors with substantial heterogeneity (Hedges *g*=−0.07, 95% CI −1.81 to 1.67; *P*=.87; *I*^2^=82%; see Figure 10). However, meta-regression or subgroup analysis was not possible due to the limited number of studies. The certainty of evidence using the GRADE approach was very low (see Multimedia Appendix 5). Although the single-arm trial by Yang et al [39] showed a reduced PNI among esophageal cancer survivors receiving adjuvant chemoradiotherapy, the reduction was significantly less than that compared to those receiving postoperative usual care [47].

## Discussion

### Principal Findings

To our best knowledge, this is the first systematic review and meta-analysis to synthesize the preliminary evidence on the effects of mobile app–based dietary interventions on anthropometric changes, nutritional outcomes, and QoL among cancer survivors. However, existing trials primarily focused on breast and GI cancer survivors. Based on these existing findings, mobile app–based dietary interventions showed potential in improving energy intake and reducing body weight among GI cancer survivors.

Our meta-analysis demonstrated that mobile app–based dietary interventions might vastly improve energy intake, specifically for GI cancer survivors. Notably, our findings on energy intake differ from a previous meta-analysis by Gong et al [17], which found no significant effects of dietary interventions on energy intake among breast, bladder, and endometrial cancer survivors using phone calls, websites, and emails. This suggests that mobile apps can be an effective alternative to other mHealth means, such as websites and emails, for improving energy intake among GI cancer survivors. Nonetheless, it is worth noting the paucity of available trials precludes definitive conclusions about whether our observed improved energy intake stems from the nature of the dietary intervention. This underscores the need for more future trials to confirm the relative contributions of dietary intervention methodology to energy intake. Furthermore, considering the effects observed in our analysis were assessed over relatively short follow-up durations (mean 4.5 months), future RCTs should evaluate the long-term impact of mobile app–based intervention on energy intake.

Notably, the WeChat app in the study by Wang et al [38] and the iNutrition applet in the study by Jiang et al [34] contributed to the pooled effect on energy intake and demonstrated high feasibility of app use among GI cancer survivors. Specifically, both studies reported attrition rates of less than 10% in the intervention groups, and the mobile app usage experience of iNutrition was rated with a good SUS score greater than 70. In contrast, another study by Huggins and colleagues [33] that also involved GI cancer survivors reported a substantially higher attrition rate (69%) in the intervention group. The differences in attrition rates may be attributed to the adoption of different dietary intervention delivery modes. Huggins and colleagues [33] solely relied on an asynchronous mode, which may have limited the immediate addressing of participants’ concerns regarding nutrition impact symptom management. Conversely, Wang et al [38] and Jiang et al [34] used both synchronous and asynchronous modes, allowing participants to choose their preferred delivery mode based on their preferred timing of mobile app use and frequency of dietitian contact via the app. Therefore, future studies targeting GI cancer survivors could explore whether such hybrid delivery modes could better address variability in participants’ needs to promote retention.

Although mobile app–based dietary interventions yielded nonsignificant effects on protein intake (Hedges *g*=1.3) and QoL (Hedges *g*=2.29), their effect sizes were relatively larger compared to other outcomes. Among the included studies, Wang and colleagues [38] showed substantially greater significant effects on protein intake and QoL than the other studies. Several reasons may explain this superior effect. First, there are differences in the clinical staging of the target population. Wang and colleagues [38] recruited stage I to III colorectal cancer survivors, while Huggins et al [33] and Jiang et al [34] included upper GI cancer patients across all clinical stages. With the inclusion of metastatic stage upper GI cancer survivors, who are more likely to experience cachexia [48], sole dietary management through mobile apps may not be sufficient to improve protein intake and QoL. Multimodal management, including pharmacotherapy, physical activity, and mental support, in addition to dietary interventions, is recommended for cachexia management according to the European Society of Medical Oncology guidelines [49]. Second, there are also differences in participants’ treatment statuses. Given nutrition impact symptoms were associated with poorer QoL among oncology patients [50], the upper GI cancer survivors in the studies by Huggins et al [33] and Jiang et al [34] were either receiving active cancer treatments or had just received gastrectomy. They may be suffering from worse nutrition impact symptoms compared to the colorectal cancer survivors in the study by Wang et al [38] who had completed active cancer treatments. Third, psychological and social functioning are essential determinants of cancer survivors’ perceived QoL [51], as measured by the global health status score of the EORTC QLQ-C30. Wang and colleagues [38] could have provided better psychological support through monthly dietitian-led dietary consultation video calls, enabling both nonverbal and verbal communication, compared to telephone and text messages, which are restricted to verbal communication. To leverage the potential of dietary mobile app–based interventions on protein intake and QoL, further trials should investigate whether cancer staging, treatment status, and the mode of communication with dietitians influence their effectiveness.

Our meta-analysis found that dietary apps may help reduce weight among GI cancer survivors. Notably, the 2 studies included, conducted by Jiang et al [34] and Huggins et al [33], did not specify their weight change goals. However, a recent systematic review has indicated that GI cancer was associated with overweight and obesity but not underweight [52]. Therefore, the weight reduction observed through the use of dietary apps can be considered beneficial. Nonetheless, additional trials with clearly defined weight loss goals are desirable to strengthen the evidence supporting the effectiveness of dietary apps in promoting favorable weight loss among GI cancer survivors.

Mobile app–based dietary interventions yielded insignificant effects on body weight in breast cancer survivors and insignificant impact on nutritional status in GI cancer survivors due to the positive and negative effects in the included studies. The mixed effects on body weight could be due to the involvement of breast cancer survivors with normal BMI and obesity, as weight loss is only indicated for those with obesity at baseline. On the other hand, the mixed effects on nutritional status could be attributed to the involvement of GI cancer survivors with different cancer treatment statuses and staging. Notably, although one of the included studies by Jiang and colleagues [34] recruited upper GI cancer survivors who may be susceptible to gastrectomy-related side effects, it still demonstrated a superior effect compared to other included studies. This may be due to the engagement of family members and caregivers during the receiving of mobile app–based dietary interventions, given both are considered an essential source of self-efficacy [34] and could have facilitated participants’ implementation of dietary management plans. Future trials may consider caregivers’ or family members’ involvement in supporting mobile app–based dietary interventions.

Only five of the 20 mobile apps reviewed in our study were evaluated on their usability, quality, and satisfaction using standard questionnaires such as the SUS, MARS, and QUIS. All users of these 5 apps, who were breast, gastric, and colorectal cancer survivors with varying treatment statuses and clinical staging, rated these apps highly in terms of usability, quality, and satisfaction. While the QUIS primarily focuses on the acceptability of computer interfaces and the SUS is not exclusively designed for mobile apps, the user version of the MARS offers a tailored evaluation of mobile apps, including engagement, functionality, aesthetics, and information quality [46]. It can be administered to individuals without expertise in mHealth [53], and also assesses the users’ perceived impact on awareness, knowledge, attitudes, and intentions toward behavioral health changes by mobile apps [46]. This aligns with the constructs of “affective attitude,” “perceived effectiveness,” “intention,” and “experience” based on the theoretical framework of acceptability for healthcare interventions [54]. Future trials could consider using the user version of the MARS for a more comprehensive and customized evaluation of mobile apps.

In over half of the studies included in our review, mobile apps incorporated feedback messages [27,40] and dietary goal setting [43], which are considered facilitators of mobile app usage. However, qualitative feedback from participants highlighted concerns related to the time required for app usage [24,26,43] and limited options for logging food entries [26,27]. To address these issues, future mobile apps could consider integrating artificial intelligence (AI)-powered food item recognition during the food logging process. This enhancement could streamline and facilitate the entry of food items, potentially reducing the time spent using the app. In addition, this approach can minimize potential recall bias associated with self-reported food intake data collected through food frequency questionnaires. Another qualitative feedback reported the significant influence of participants’ knowledge on their intention to use mobile apps [34]. Considering the lack of cancer-specific dietary recommendations in the mobile apps examined in our review, future trials could consider incorporating education on the summary of evidence regarding the association between dietary patterns and cancer risks provided by the World Cancer Research Fund International and the American Institute for Cancer Research.

### Strengths and Limitations

A strength of this study is that the systematic search of this review was not limited by language, and there were no restrictions on the cancer types, treatment statuses, prognoses, weight statuses, and age of our target population. However, several limitations should be acknowledged. First, most of the included studies comprised a small number of participants and were quasi-experimental in nature. To enhance the robustness of our findings, we conducted meta-analyses only on outcomes of interest using postintervention means and SDs from RCTs. Future trials with larger sample sizes are needed to further validate the efficacy of these interventions. Second, despite the high study heterogeneity among studies, conducting subgroup analyses or meta-regressions to examine relevant moderators by study, intervention, and participants’ characteristics was not feasible due to the limited number of available trials. In addition, the diverse combinations of app features across studies hindered the identification of specific features that were more effective in promoting the desired outcomes. Future research could isolate specific app features to explore their individual contributions. Third, the studies included in the meta-analyses did not specify weight change goals, leaving uncertainty about whether the observed weight changes were in a favorable direction. However, we attempted to stratify the intervention effect by cancer type and by weight status as determined by the mean baseline BMI. All results on weight and BMI remained the same. Future studies should specify weight change goals to better determine the direction of favorable change. Fourth, the meta-analysis included only a limited number of studies. It would be beneficial to have more research, particularly on effects that are currently considered insignificant but may have potential, such as protein intake. Fifth, the studies incorporated into the meta-analysis focused solely on survivors of breast or GI cancer, which means our findings may not be generalized to survivors of other types of cancer.

### Conclusions

In conclusion, this systematic review and meta-analysis provide preliminary evidence for the potential efficacy, feasibility, and acceptability of dietary interventions delivered through mobile apps for cancer survivors. While the existing evidence needs to be strengthened, dietary mobile apps can have a positive impact on energy intake and weight changes among GI cancer survivors. The usability, quality, or satisfaction of all mobile apps evaluated by standardized questionnaires was rated highly by breast, gastric, or colorectal cancer survivors with varying treatment statuses and clinical staging. To optimize the effectiveness of mobile apps for cancer survivors, future mobile apps could retain essential features such as feedback messages and dietary goal setting while incorporating new features such as AI-powered food item recognition during food logging and cancer-specific dietary recommendations. Further trials are necessary to determine whether the effectiveness of intervention varies based on cancer types, staging, treatment status, the mode of communication with dietitians, and the engagement of family or caregivers.

## Supplementary material

10.2196/65505 Multimedia Appendix 1 Search strategy.

10.2196/65505 Multimedia Appendix 2 Additional study characteristics.

10.2196/65505 Multimedia Appendix 3 Risk of bias assessments.

10.2196/65505 Multimedia Appendix 4 Summary of usability, quality, or satisfaction of mobile app use.

10.2196/65505 Multimedia Appendix 5 Grading of Recommendations Assessment, Development, and Evaluation (GRADE) assessment.

10.2196/65505 Checklist 1 PRISMA (Preferred Reporting Items for Systematic Reviews and Meta-Analyses) 2020 checklist.

## Abbreviations

AI: artificial intelligence
GI: gastrointestinal
GRADE: Grading of Recommendations Assessment, Development and Evaluation
MARS: Mobile Application Rating Scale
mHealth: mobile health
PG-SGA: Scored Patient-Generated Subjective Global Assessment
PNI: prognostic nutritional index
PRISMA: Preferred Reporting Items for Systematic Reviews and Meta-Analyses
QLQ-C30: European Organization for the Research and Treatment of Cancer Quality of Life Questionnaire
QoL: quality of life
QUIS: Questionnaire for User Interface Satisfaction
RCT: randomized controlled trial
RoB: Risk of bias
ROBINS-I: Risk of Bias in Nonrandomized Studies
SMD: standardized mean difference
uMARS: user version of the Mobile Application Rating Scale
