# Supplementary material for: Efficacy of Mobile App–Based Dietary Interventions Among Cancer Survivors: Systematic Review and Meta-Analysis
Source: JMIR Mhealth Uhealth. 2025 Jul 31;13:e65505. doi: 10.2196/65505 (PMC12312991; doi:10.2196/65505)
Supplement: Multimedia Appendix 1 [file mhealth-v13-e65505-s001.docx]

**Search strategy of each database**

**EMBASE**

1. cancer.ab,ti
2. oncology.ab,ti.
3. tumour.ab,ti.
4. tumor.ab,ti.
5. malignan*.ab,ti.
6. 1 or 2 or 3 or 4 or 5
7. diet*.ab,ti.
8. nutrition*.ab,ti.
9. behavior*.ab,ti.
10. behaviour*.ab,ti.
11. 7 or 8 or 9 or 10
12. mobile app*.ab,ti.
13. mhealth.ab,ti.
14. smartphone app*.ab,ti.
15. e-health.ab,ti.
16. mobile-assisted.ab,ti.
17. technology-supported.ab,ti.
18. app.ab,ti.
19. 12 or 13 or 14 or 15 or 16 or 17 or 18
20. 6 and 11 and 19

**Cochrane library**

#1 (cancer):ti,ab,kw OR (oncology):ti,ab,kw OR (tumour):ti,ab,kw OR (tumor):ti,ab,kw OR (malignan*):ti,ab,kw

#2 (diet*):ti,ab,kw OR (nutrition*):ti,ab,kw OR (behavior*):ti,ab,kw OR (behaviour*):ti,ab,kw

#3 (mobile app*):ti,ab,kw OR (mhealth):ti,ab,kw OR (smartphone app*):ti,ab,kw OR (mobile-assisted):ti,ab,kw OR (technology-supported):ti,ab,kw OR (app):ti,ab,kw OR (e-health):ti,ab,kw

#4 (#1) AND (#2) AND (#3)

**Pubmed**

((cancer[Title/Abstract]) OR (oncology[Title/Abstract]) OR (tumour[Title/Abstract]) OR (tumor[Title/Abstract]) OR (malignan*[Title/Abstract])) AND ((diet*[Title/Abstract]) OR (nutrition*[Title/Abstract]) OR (behavior*[Title/Abstract]) OR (behaviour*[Title/Abstract])) AND ((mobile app*[Title/Abstract]) OR (mhealth[Title/Abstract]) OR (smartphone app* [Title/Abstract]) OR (mobile-assisted[Title/Abstract]) OR (technology-supported[Title/Abstract]) OR (app[Title/Abstract]) OR (e-health[Title/Abstract]))

**Web of Science**

(TI=(cancer) OR TI=(oncology) OR TI=(tumour) OR TI=(tumor) or TI=(malignan*)) AND (TI=(diet*) OR TI=(nutrition*) OR TI=(behaviour*) OR TI=(behavior*)) AND (TI=(mobile app*) OR TI=(mhealth) OR TI=(smartphone app*) OR TI=(mobile-assisted) OR TI=(technology-supported) OR TI=(app*) OR TI=(e-health))
