# Supplementary material for: Efficacy of Mobile App–Based Dietary Interventions Among Cancer Survivors: Systematic Review and Meta-Analysis
Source: JMIR Mhealth Uhealth. 2025 Jul 31;13:e65505. doi: 10.2196/65505 (PMC12312991; doi:10.2196/65505)
Supplement: Multimedia Appendix 2 [file mhealth-v13-e65505-s002.docx]

**Additional study characteristics**

| **Citation, Study design, Country** | **Length of follow-up (attrition rate)** | **Outcomes** | **Mean change from baseline**  **IG^a^ (vs CG^b^, if available)** | **Post-intervention means of IG (vs CG, if available)** |
| --- | --- | --- | --- | --- |
| Allicock et al. (2021)  Mixed method  RCT^c^; The United States | 8 weeks  (no attrition) | 1. Weight (lbs) 2. BMI^d^ (kg/m^2^) 3. Fast food intake (servings/day) 4. Fruit and vegetable intake (servings/day) | N/A^e^ | At 4 weeks:   1. 206.85 (SE^f^=0.98) vs 207.10 (SE=1.15) (*P*=0.909) 2. 35.38 (SE=NR) vs 35.44 (SE=NR) (*P*=0.803) 3. 3.46 (SE=0.46) vs 3.61 (SE=0.53) (*P*=0.834) 4. 6.33 (SE=0.67) vs 6.44 (SE=0.77) (*P*=0.914)   At 8 weeks:   1. 206.53 (SE=1.47) vs 204.03 (SE=1.92) (*P*=0.336) 2. 35.06 (SE=0.24) vs 34.79 (SE=0.31) (*P*=0.514) 3. 3.03 (SE=0.59) vs 3.57 (SE=0.76) (*P*=0.581) 4. 5.93 (SE=0.39) vs 6.48 (SE=0.50) (*P*=0.403) |
| Baik et al. (2020)  RCT; The United States (Latina) | 8 weeks  (Both IG and CG: 2.5%) | FACT-B^g^ subscales   1. Emotional well-being 2. Functional well-being 3. Physical well-being 4. Social well-being | N/A | At 6 weeks:  High app^h^ users (≥ 60 minutes/week)   1. 19.09 (SD^i^=NR^j^) vs 18.33 (SD=NR) (*P*=NR) 2. 20.55 (SD=NR) vs 21.33 (SD=NR) (*P*=NR) 3. 21.27 (SD=NR) vs 21.56 (SD=NR) (*P*=NR) 4. 21.15 (SD=NR) vs 23.40 (SD=NR) (*P*=NR)   Low app users (< 60 minutes/week)   1. 19.67 (SD=NR) vs 18.95 (SD=NR) (*P*=NR) 2. 20.13 (SD=NR) vs 20.55 (SD=NR) (*P*=NR) 3. 23.20 (SD=NR) vs 21.10 (SD=NR) (*P*=NR) 4. 20.44 (SD=NR) vs 21.64 (SD=NR) (*P*=NR)   At 8 weeks:  High app users (≥ 60 minutes/week)  1. 18.60 (SD=NR) vs 19.00 (SD=NR) (*P*=NR)  2. 19.95 (SD=NR) vs 21.06 (SD=NR) (*P*=NR)  3. 20.27 (SD=NR) vs 20.00 (SD=NR) (*P*=NR)  4. 20.90 (SD=NR) vs 22.52 (SD=NR) (*P*=NR)  Low app users (< 60 minutes/week)   1. 18.87 (SD=NR) vs 17.68 (SD=NR) (*P*=NR) 2. 19.47 (SD=NR) vs 20.95 (SD=NR) (*P*=NR) 3. 21.80 (SD=NR) vs 21.00 (SD=NR) (*P*=NR) 4. 19.87 (SD=NR) vs 21.23 (SD=NR) (*P*=NR) |
| Cairo et al. (2020)  Mixed method quasi-experimental study; The United States | 48 weeks  (13% of all subjects at the end of the 6-month intervention; 100% among 41 subjects who completed the questionnaire at 12 months) | 1. Weight (kg) 2. BMI (kg/m^2^) | N/A | At 24 weeks:   1. 79.7 (SD=19.2) vs 84.4 (SD=21.7) (*P*=0.20) 2. 28.7 (SD=5.9) vs 30.3 (SD=7.4) (*P*=0.18) |
| Cheng et al. (2020)  Mixed method single-arm trial; China | 12 weeks (0% after 4 weeks; 20% after 12 weeks) | 1. EORTC QLQ-C30^k^ global health status scores 2. Weight (kg) 3. BMI (kg/m^2^) | At 4 weeks:   1. -11.3 (*P*=0.02) 2. -3.7 (*P*<0.001) 3. -1.0 (*P*=0.02)   At 12 weeks:   1. +4.4 (*P*≥0.05) 2. -1.3 (*P*<0.001) 3. -0.50 (*P*=0.02) | N/A |
| Choi et al. (2020)  RCT; South Korea | 4 weeks  (IG: 16%; CG: 12%) | 1. Vegetable intake (weekly servings) 2. Fruit intake (weekly servings) 3. Fast food intake (weekly servings) 4. Weight (kg) 5. BMI (kg/m^2^) 6. Program satisfaction survey scores (range 0-5) | N/A | At 4 weeks:   1. 5.7 (SD=4.6) vs 3.7 (SD=1.4) (*P*=0.003) 2. 7.9 (SD=4.3) vs 6.7 (SD=3.8) (*P*=0.004) 3. 0.8 (SD=0.5) vs 0.9 (SD=0.7) (*P*=0.037) 4. 62.2 (SD=8.9) vs 59.1 (SD=9.2) (*P*≥0.05) 5. 24.1 (SD=4.1) vs 23.4 (SD=3.2) (*P*≥0.05) 6. 4.2 |
| Chow et al. (2021)  Mixed method RCT; The United States | 24 weeks (9.8% for all subjects in this study) | 1. Added sugar intake (Daily intake %) 2. PROMIS^l^ Global 10 t-scores | At 16 weeks:   1. -0.8% (95%CI^m^ -2.2, 0.5) vs +0.1% (95%CI -1.5, 1.6) (*P*=0.39) 2. Physical health subscale   +2.7 (95%CI 0.7, 4.6) vs +1.8 (95%CI -0.3, 3.8) (*P*=0.52)  Mental health subscale  +4.2 (95%CI 1.5, 6.9) vs +1.8 (95%CI -1.1, 4.8) (*P*=0.24) | N/A |
| Fuemmeler et al. (2020)  Single-arm trial, The United States | 8 weeks  (6.3%) | 1. Energy intake (kcal/day)  2. Fruit and vegetable intake (servings/day)  3. Energy intake from sweet foods (% of total energy intake)  4. Sugary drinks consumption (g/day)  5. BMI (kg/m^2^) | At 8 weeks:   1. -35.1 (*P*=0.693) 2. -0.1 (*P*=0.176) 3. -2.1 (*P*=0.451) 4. -10.9 (*P*=0.164) 5. +0.4 (*P*=0.226) | N/A |
| Huggins et al. (2022)  3-arm RCT; Australia | 48 weeks  (MIG^n^: 69%; TIG^o^: 58%; CG: 86%) | 1. EORTC QLQ-C30 global health status scores 2. PG-SGA_SF_^p^ scores 3. Weight (kg) | N/A | MIG vs TIG vs CG at 24 weeks:   1. 59.25 (SD=21.10) vs 68.0 (SD=28.13) vs 69.8 (SD=12.2) 2. 7.2 (SD=4.0) vs 6.2 (SD=5.1) vs 4.6 (SD=3.6) 3. 68.7 (SD=14.1) vs 70.2 (SD=11.7) vs 75.6 (SD=17.5)   MIG vs TIG vs CG at 48 weeks:   1. 73.5 (SD=20.5) vs 74.8 (SD=23.8) vs 72.7 (SD=15.9) 2. 4.9 (SD=3.6) vs 4.3 (SD=4.7) vs 4.1 (SD=4.1) 3. 68.5 (SD=14.1) vs 68.6 (SD=13.3) vs 73.2 (SD=18.4) |
| Jiang et al. (2023)  Mixed method RCT; China | 12 weeks  (IG: 8%; CG: 17%) | 1. PG-SGA^q^ scores 2. Weight (kg) 3. BMI (kg/m^2^) 4. Energy intake (kcal/day) 5. Protein intake (kcal/day) 6. EORTC QLQ-C30 global health status scores 7. SUS^r^ scores (each item on a 5-point Likert scale; total score computed to: 0-100) 8. NPS^s^ scores (dividing % of detractors by % of promoters; -100% to +100%) | N/A | At 12 weeks:   1. 5.50 (SE=0.85) vs 7.50 (SE=0.86) (*P*=0.379) 2. 55.43 (SE=2.38) vs 59.50 (SE=2.84) (*P*=0.094) 3. 20.57 (SE=0.81) vs 21.77 (SE=0.73) (*P*=0.066) 4. 1072.52 (SE=86.94) vs 811.72 (SE=56.64) (*P*=0.011) 5. 53.83 (SE=6.33) vs 38.68 (SE=3.20) (*P*=0.046) 6. 75.61 (SE=3.45) vs 68.48 (SE=3.09) (*P*=0.847) 7. Mean total 77.27 (SD=10.69) 8. +18.2% |
| Keum et al. (2021)  RCT; South Korea | 12 weeks  (IG: 15%; CG: 20%) | 1. EORTC QLQ-C30 global health status scores 2. PG-SGA scores 3. Protein intake (kg/day) 4. Energy intake (kcal/kg/day) | N/A | At 12 weeks:   1. Approximately 70 (SD=NR) vs 45 (SD=NR) (*P*=0.004) 2. Approximately 9.5 (SD=NR) vs 10.5 (SD=NR) (*P*=NR)   Users with app use >9 weeks vs users with app use <9 weeks  3. 1.3 vs 1.0 (*P*=0.02)  4. 25.2 vs 17.7 (*P*=0.04) |
| Lim et al. (2023)  Single-arm study; South Korea | 48 weeks  (38%) | 1. Needs and satisfaction scores (0-30) 2. Usability (14-item usability scale on 5-point Likert scale but converted to 0-100) | N/A | At 48 weeks:   1. mean total 24.6 (SD=0.1) and 22.4 (SD=0.2) 2. mean total 80.2 (SD=7.9) |
| Lozano et al. (2019)  Mixed method single-arm trial; Spain | 8 weeks  (5%) | 1. EORTC-QLQ-C30 global health status scores 2. Weight (kg) 3. BMI (kg/m^2^) 4. MARS^t^ score (1-5) 5. NPS scores (-100 to +100) | At 8 weeks:   1. +12.83 (95%CI 8.95, 16.71; *P*<0.001) 2. -1.42 (95%CI -1.97, -0.86; *P*<0.001) 3. -0.57 (95%CI -0.81, -0.34; *P*<0.001) | At 8 weeks:   1. overall mean 3.71 (SD=0.47) 2. +6.58 |
| McCarroll et al. (2015)  Single-arm trial; The United States | 4 weeks  (30%) | 1. Energy intake (kcal/week) 2. Protein intake (g/week) 3. Weight (kg) 4. BMI (kg/m^2^) 5. FACT-G^u^ scores | At 4 weeks:   1. +258.5 (SD=NR) (*P*=0.263) 2. +10.2 (SD=NR) (*P*=0.226) 3. -2.3 (SD=NR) (*P*<0.001) 4. -0.8 (SD=NR) (*P*<0.001) 5. -6.0 (95%CI -14.3, 2.3) (*P*=0.152) | N/A |
| Orlemann et al. (2018)  Mixed method quasi-experimental study; Germany | 4 weeks  (38% in total prior to the start of the study) | Weight (kg) | At 4 weeks:  +1.03 vs -1.46 (p=0.045) | N/A |
| Park et al. (2019)  Quasi-experimental study; South Korea | 24 weeks  (IG: 61% with 42% due to dropouts prior to intervention; CG: 37%) | 1. Energy intake (kcal/day) 2. Vegetable intake (g/day) 3. BMI (kg/m^2^) | N/A | At 12 weeks:   1. 1368.78 (SD=208.53) vs 1627.52 (SD=413.60) (*P*=NR) 2. 455.62 (SD=189.01) vs 349.47 (SD=95.52) (*P*=NR) 3. 25.64 (SD=2.72) vs 26.64 SD=2.74) (*P*<0.001)   At 24 weeks:   1. 1485.81 (SD=195.69) vs 1600.31 (SD=292.30) (*P*=0.163) 2. 378.06 (SD=187.56) vs 348.63 (SD=140.62) (*P*=0.342) 3. 25.86 (SD=2.76) vs 26.96 (SD=2.87) (*P*<0.001) |
| Salmani et al. (2022)  Single-arm trial; Iran | 2 weeks  (No attrition) | QUIS^v^ scores (0-9) | N/A | At 2 weeks:  Overall reaction to the app  7.94 (SD=1.38)  Screen design and layout  8.18 (SD=1.17)  Terminology and app information  7.97 (SD=1.27)  Learnability  7.98 (SD=1.25)  App features  9.12 (SD=1.31) |
| Seo et al. (2021)  Single-arm trial; South Korea | 2 weeks  (No attrition) | User version of MARS scores (1-5) | N/A | At 2 weeks:  Mean total 3.60 (SD=0.69) |
| Soh et al. (2018)  Single-arm trial;  South Korea | 12 weeks  (16% and 11% for gastric and colon cancer subjects respectively) | Satisfaction scores (5-point Likert scale) | N/A | At 12 weeks:  Overall score between 3.93 (SD=0.88) and 4.01 (SD=0.87) |
| Stubbins et al. (2018)  Single-arm trial; The United States | 4 weeks  (19%) | 1. Weight (lbs) 2. SUS scores (each item on a 5-point Likert scale; total score computed to: 0-100) | At 4 weeks:   1. -2 (SD=NR) | At 4 weeks:   1. Mean total 77.4 (SD=NR) |
| Wang et al. (2022)  RCT; China | 24 weeks  (Both IG and CG: 6%) | 1. Weight (kg) 2. BMI (kg/m^2^) 3. PG-SGA scores 4. Energy intake (kcal/day) 5. Protein intake (g/day) 6. EORTC QLQ-C30 global health status scores | At 24 weeks:   1. +2.00 (95%CI 0.25, 3.00) vs 0 (95%CI -1.75, 0) (*P*<0.001) | At 24 weeks:   1. 24.60 (SD=2.71) vs 23.48 (SD=3.07) (*P*=0.155) 2. 3 (95%CI 2, 4) vs 4.5 (95%CI 3, 7) (*P* =0.010) 3. 1,602.38 (SD=148.95) vs 1,397.45 (SD=244.88) (*P* =0.001) 4. 78.80 (SD =16.99) vs 53.46 (SD=13.09) (*P* <0.001) 5. 83.93 (SD=3.15) vs 54.17 (SD=5.04) (*P* <0.001) |
| Yang et al. (2021)  Single-arm trial; South Korea | 8 weeks  (5%) | Prognostic nutritional index | After 5 weeks of radiotherapy:  -6.7 (SD=7.5) vs -9.8 (SD=6.0) | N/A |
| Yang et al. (2022)  Single-arm trial; South Korea | 12 weeks  (16%) | 1. EORTC QLQ-C30 global health status scores 2. Smart After-Care Program satisfaction survey scores | Median change from baseline after 12 weeks:   1. +16.6 (*P*<0.001) | After 12 weeks:   1. Overall satisfaction   88% rated “very good” or “good” |

^a^IG: intervention group.

^b^CG: control group.

^c^RCT: randomised controlled trial.

^d^BMI: body mass index.

^e^N/A: not applicable.

^f^SE: standard error.

^g^FACT-B: Functional Assessment of Cancer Therapy – Breast.

^h^app: application.

^i^SD: standard deviation.

^j^NR: not reported.

^k^EORTC QLQ-C30: European Organization for the Research and Treatment of Cancer Core Quality of Life Questionnaire.

^l^PROMIS: Patient-Reported Outcomes Measurement Information System.

^m^CI: confidence interval.

^n^MIG: myPace app intervention group.

^o^TIG: telephone intervention group.

^p^PG-SGA_SF_: The Scored Patient-Generated Subjective Global Assessment Short Form.

^q^PG-SGA: The Scored Patient-Generated Subjective Global Assessment.

^r^SUS: System Usability Scale.

^s^NPS: Net Promoter Score.

^t^MARS: Mobile Application Rating Scale.

^u^FACT-G: Functional Assessment of Cancer Therapy – General.

^v^QUIS: Questionnaire for User Interaction Satisfaction.
