# Supplementary material for: Efficacy of Mobile App–Based Dietary Interventions Among Cancer Survivors: Systematic Review and Meta-Analysis
Source: JMIR Mhealth Uhealth. 2025 Jul 31;13:e65505. doi: 10.2196/65505 (PMC12312991; doi:10.2196/65505)

**Risk of bias assessments of randomised controlled trials**


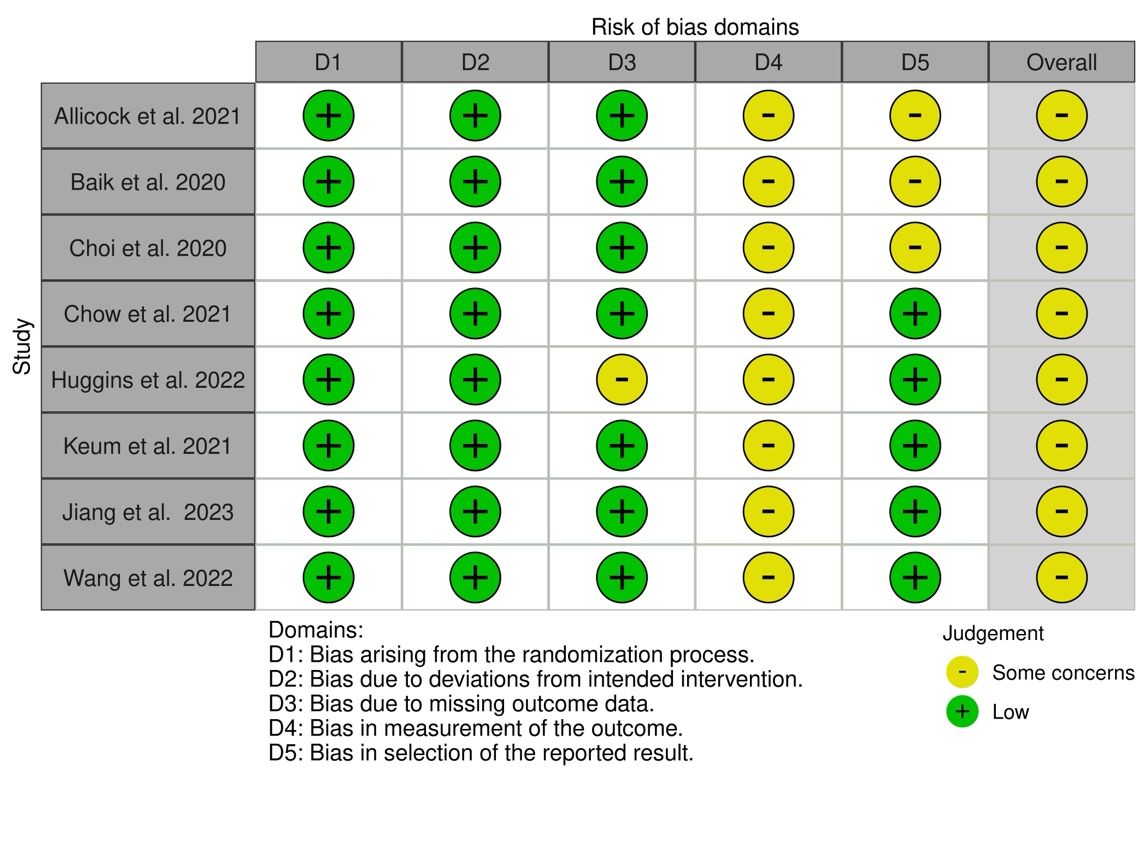


**Risk of bias assessments of non-randomised trials**


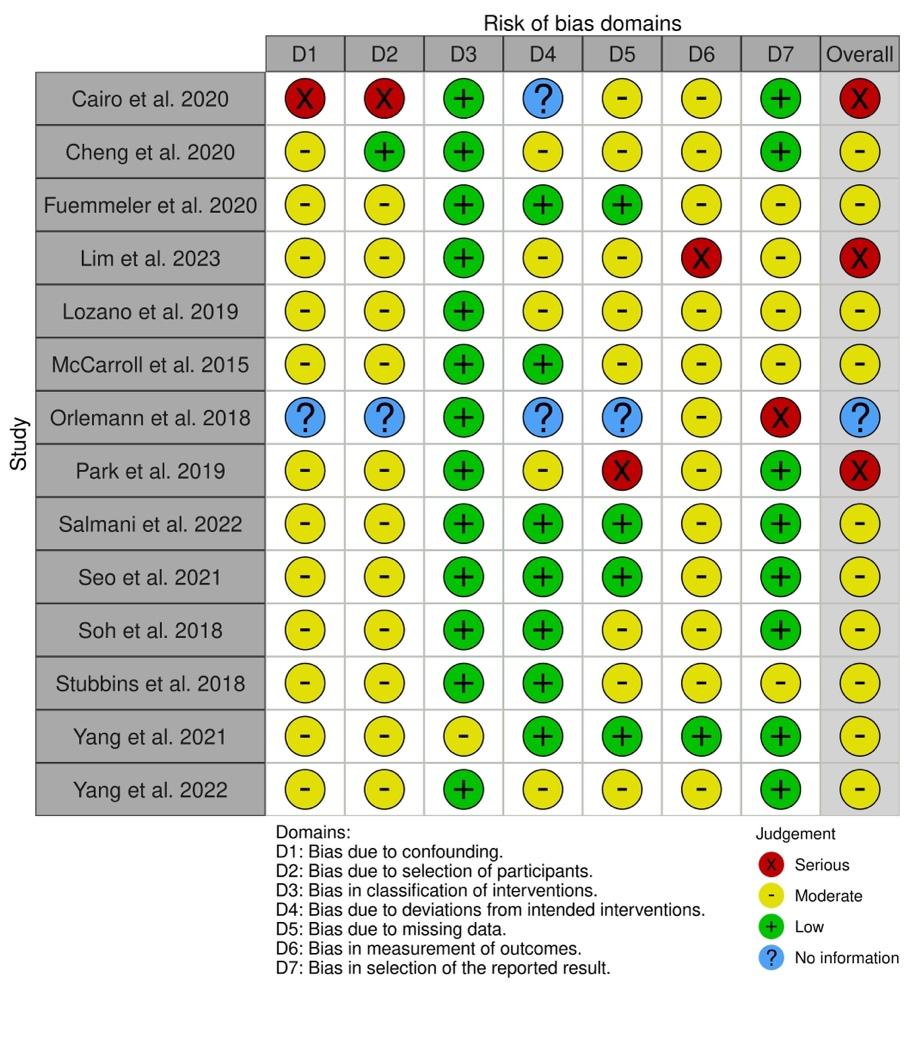

Supplement: Multimedia Appendix 3 [file mhealth-v13-e65505-s003.docx]
