# Supplementary material for: Efficacy of Mobile App–Based Dietary Interventions Among Cancer Survivors: Systematic Review and Meta-Analysis
Source: JMIR Mhealth Uhealth. 2025 Jul 31;13:e65505. doi: 10.2196/65505 (PMC12312991; doi:10.2196/65505)
Supplement: Multimedia Appendix 4 [file mhealth-v13-e65505-s004.docx]

**Summary of usability, quality, or satisfaction of mobile app use**

| **Mobile application name, Citation, Study design, Country** | **Sample characteristics** | **Outcomes** | **Post-intervention means of IG^a^ (vs CG^b^, if available)** |
| --- | --- | --- | --- |
| efilcare R app^c^  Choi et al. (2020)  RCT^d^; South Korea | - Breast cancer  - Mean age: 49 years  - Female: 100%  - Mean BMI^e^: 23.9 kg/m^2^  - Staging: NR^f^  - Completed cancer treatments | Program satisfaction survey scores (range 0-5) | At 4 weeks:  4.2 |
| iNutrition applet  Jiang et al. (2023)  Mixed method RCT; China | - Gastric cancer  - Mean age: 55 years  - Female: 33%  - Mean BMI: 23.6 kg/m^2^  - Stage I-IV  - Post-discharge after gastrectomy | 1. SUS^g^ scores (each item on a 5-point Likert scale; total score computed to: 0-100) 2. NPS^h^ scores (dividing % of detractors by % of promoters; -100% to +100%) | At 12 weeks:   1. Mean total 77.27 (SD^i^=10.69) 2. +18.2% |
| Second Doctor app  Lim et al. (2023)  Single-arm study; South Korea | - Breast cancer  - Mean age: 43 years  - Female: 100%  - Mean BMI: NR  - Stage 0-IIB  - Just received surgical treatment and pending chemo-, radio- or hormonal therapy | 1. Needs and satisfaction scores (0-30) 2. Usability (14-item usability scale on 5-point Likert scale but converted to 0-100) | At 48 weeks:   1. mean total 24.6 (SD=0.1) and 22.4 (SD=0.2) 2. mean total 80.2 (SD=7.9) |
| BENECA^j^ app  Lozano et al. (2019)  Mixed method single-arm trial; Spain | - Breast cancer  - Mean age: 52 years  - Female: 100%  - BMI > 25 kg/m^2^  - Stage I to IIIA  - Completed adjuvant treatments at least half year before | 1. MARS^k^ score (1-5) 2. NPS scores (-100 to +100) | At 8 weeks:   1. overall mean 3.71 (SD=0.47) 2. +6.58 |
| “Colorectal Cancer Along” app  Salmani et al. (2022)  Single-arm trial; Iran | - Colorectal cancer  - Mean age: 58 years  - Female: 41%  - Mean BMI: NR  - Staging: NR  - Receiving cancer treatments | QUIS^l^ scores (0-9) | At 2 weeks:  Overall reaction to the app  7.94 (SD=1.38)  Screen design and layout  8.18 (SD=1.17)  Terminology and app information  7.97 (SD=1.27)  Learnability  7.98 (SD=1.25)  App features  9.12 (SD=1.31) |
| “Health for You” app  Seo et al. (2021)  Single-arm trial; South Korea | - Breast cancer  - Mean age: 51 years  - Female: 100%  - Mean BMI: 26.8 kg/m^2^  - Stage I-III  - Completed cancer treatments | User version of MARS scores (1-5) | At 2 weeks:  Mean total 3.60 (SD=0.69) |
| Life Manager app  Soh et al. (2018)  Single-arm trial;  South Korea | - Gastric (49.8%) or colon cancer  - Mean age: NR (35.5% in their 50s)  - Female: 30% gastric; 44% colon  - Mean BMI: NR  - Staging: NR  - Received surgery or receiving chemotherapy (50.2% receiving palliative care) | Satisfaction scores (5-point Likert scale) | At 12 weeks:  Overall score between 3.93 (SD=0.88) and 4.01 (SD=0.87) |
| MOCHA^m^ app  Stubbins et al. (2018)  Single-arm trial; The United States | - Breast cancer  - Mean age: 57 years  - Female: 100%  - Mean BMI: 31.6 kg/m^2^  - Stage I to III  - Completed cancer treatments | SUS scores (each item on a 5-point Likert scale; total score computed to: 0-100) | At 4 weeks:  Mean total 77.4 (SD=NR) |
| Smart After-Care app  Yang et al. (2022)  Single-arm trial; South Korea | - Lung cancer  - Female: 44%  - Mean age: 58 years  - Mean BMI: 23.4 kg/m^2^  - Stage I to IV  - Receiving outpatient chemotherapy treatments or paying routine outpatient visits after lung resection surgery | Smart After-Care Program satisfaction survey scores | At 12 weeks:  Overall satisfaction  88% rated “very good” or “good” |

^a^IG: intervention group.

^b^CG: control group.

^c^app: application.

^d^RCT: randomised controlled trial.

^e^BMI: body mass index.

^f^NR: not reported.

^g^SUS: System Usability Scale.

^h^NPS: Net Promoter Score.

^i^SD: standard deviation.

^j^BENECA: The Energy Balance on Cancer.

^k^MARS: Mobile Application Rating Scale.

^l^QUIS: Questionnaire for User Interaction Satisfaction.

^m^MOCHA: Methodist Hospital Cancer Health Application.
