# Supplementary material for: Efficacy of Mobile App–Based Dietary Interventions Among Cancer Survivors: Systematic Review and Meta-Analysis
Source: JMIR Mhealth Uhealth. 2025 Jul 31;13:e65505. doi: 10.2196/65505 (PMC12312991; doi:10.2196/65505)
Supplement: Multimedia Appendix 5 [file mhealth-v13-e65505-s005.docx]

**GRADE assessment**

| Certainty assessment | | | | | | | Number of cancer survivors | | Effect (95%CI) | Certainty |
| --- | --- | --- | --- | --- | --- | --- | --- | --- | --- | --- |
| Number of studies | Study design | Risk of bias | Inconsistency | Indirectness | Imprecision | Other considerations | Mobile app group | Control group |  |  |
| Energy intake (follow-up: 3 to 6 months) | | | | | | | | | | |
| 2 [1,2] | Randomised controlled trials | not serious | not serious | not serious | not serious^a^ | none | 40 | 40 | SMD 1.00 (0.96, 1.03) | 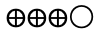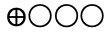  High |
| Protein intake (follow-up: 3 to 6 months) | | | | | | | | | | |
| 2 [1,2] | Randomised controlled trials | not serious | not serious^b^ | not serious | very serious^c^ | large effect size | 40 | 40 | SMD 1.30 (-3.77, 6.37) | 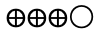  Moderate |
| Nutritional status (follow-up: 3 to 12 months) | | | | | | | | | | |
| 2 [1-3] | Randomised controlled trials | not serious | very serious^d^ | not serious | very serious^e^ | none | 65 | 77 | SMD -0.07 (-1.81, 1.67) | 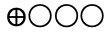  Very low |
| Body weight [Breast cancer] (follow-up: 1 to 2 months) | | | | | | | | | | |
| 2 [4,5] | Randomised controlled trials | not serious | not serious | not serious | very serious^f^ | none | 34 | 31 | SMD -0.16 (-0.76, 0.45) | 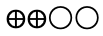  Low |
| Body weight [Gastrointestinal cancer] (follow-up: 3 to 12 months) | | | | | | | | | | |
| 2 [1,3] | Randomised controlled trials | not serious | not serious | not serious | not serious^g^ | none | 48 | 49 | SMD -0.43 (-0.45, -0.41) | 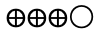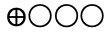  High |
| Body mass index [Breast cancer] (follow-up: 1 to 2 months) | | | | | | | | | | |
| 2 [4,5] | Randomised controlled trials | not serious | serious^h^ | not serious | very serious^i^ | none | 34 | 31 | SMD -0.06 (-0.88, 0.76) | 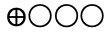  Very low |
| Body mass index [Gastrointestinal cancer] (follow-up: 3 to 6 months) | | | | | | | | | | |
| 2 [1,2] | Randomised controlled trials | not serious | serious^j^ | not serious | very serious^k^ | none | 40 | 40 | SMD 0.03 (-5.09, 5.16) | 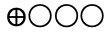  Very low |
| Quality of life (follow-up: 3 to 12 months) | | | | | | | | | | |
| 3 [1-3] | Randomised controlled trials | not serious | very serious^l^ | not serious | very serious^m^ | large effect size | 76 | 77 | SMD 2.29 (-7.80, 12.38) | 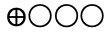  Very low |
| Fast food intake (follow-up: 1 to 2 months) | | | | | | | | | | |
| 2 [4,5] | Randomised controlled trials | not serious | not serious | not serious | very serious^n^ | none | 34 | 31 | SMD -0.14 (-0.57, 0.30) | 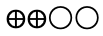  Low |

**SMD**: standardised mean difference. **CI**: confidence interval.

***Explanations***

1. Not downgraded for imprecision. Although the total sample size was low (<400 events), the confidence interval did not cross the line of null effect.
2. Not downgraded for inconsistency. Although the I^2^ was 57%, the p-value for heterogeneity was 0.13 and the 95% CI overlaps mainly between studies.
3. Downgraded by two levels for imprecision since the total sample size was low (<400 events) and the confidence interval spanned a large negative and a very large positive effect.
4. Downgraded by two levels for inconsistency since I^2^ was 81%, the p-value for heterogeneity was <0.01. The direction of effect was inconsistent. Subgroup analyses were not possible due to the low number of studies.
5. Downgraded by two levels for imprecision since the total sample size was low (<400 events) and the confidence interval spanned a large positive and negative effect.
6. Downgraded by two levels for imprecision since the total sample size was low (<400 events) and the confidence interval spanned a moderate-to-large negative and a small-to-moderate positive effect.
7. Not downgraded for imprecision. Although the total sample size was low (<400 events), the confidence interval did not cross the line of null effect.
8. Downgraded by one level for inconsistency since I^2^ was 66% and the direction of effect was inconsistent. Subgroup analyses were not possible due to the low number of studies.
9. Downgraded by two levels for imprecision since the total sample size was low (<400 events) and the confidence interval spanned a large negative and moderate-to-large positive effect.
10. Downgraded by one level for inconsistency since I^2^ was 63% and the direction of effect was inconsistent. Subgroup analyses were not possible due to the low number of studies.
11. Downgraded by two levels for imprecision since the total sample size was low (<400) and the confidence interval spanned a very large negative and positive effect.
12. Downgraded by two levels for inconsistency since I^2^ was 98%, the p-value for heterogeneity was <0.01. The direction of effect was inconsistent. Subgroup analyses were not possible due to the low number of studies.
13. Downgraded by two levels for imprecision since the total sample size was low (<400) and the confidence interval spanned a very large negative and positive effect.
14. Downgraded by two levels for imprecision since the total sample size was low (<400) and the confidence interval spanned a moderate negative and small-to-moderate positive effect.

**References**

1. Jiang X, Chen J, Yuan X, Lin Y, Chen Y, Li S, et al. Feasibility of an Individualized mHealth Nutrition (iNutrition) Intervention for Post-Discharged Gastric Cancer Patients Following Gastrectomy: A Randomized Controlled Pilot Trial. Nutrients. 2023;15(8).

2. Wang X, Zeng H, Li L, Fang Z, Xu L, Shi W, et al. Personalized nutrition intervention improves nutritional status and quality of life of colorectal cancer survivors in the community: A randomized controlled trial. Nutrition. 2022;103-104:111835.

3. Huggins CE, Hanna L, Furness K, Silvers MA, Savva J, Frawley H, et al. Effect of Early and Intensive Telephone or Electronic Nutrition Counselling Delivered to People with Upper Gastrointestinal Cancer on Quality of Life: A Three-Arm Randomised Controlled Trial. Nutrients. 2022;14(15).

4. Allicock M, Kendzor D, Sedory A, Gabriel KP, Swartz MD, Thomas P, et al. A Pilot and Feasibility Mobile Health Intervention to Support Healthy Behaviors in African American Breast Cancer Survivors. J Racial Ethn Health Disparities. 2021;8(1):157-65.

5. Choi JH, Park S-J, Kwon H, Lee H-J. Application and evaluation of mobile nutrition management service for breast cancer patients. jnh. 2020;53(1):83-97.
